# Hepatoprotective effects of bioactive compounds from traditional herb Tulsi (*Ocimum sanctum* Linn) against galactosamine-induced hepatotoxicity in rats

**DOI:** 10.3389/fphar.2023.1213052

**Published:** 2023-10-04

**Authors:** Fatemah O. Kamel, Shahid Karim, Duaa Abdullah Omer Bafail, Hibah Mubarak Aldawsari, Sabna Kotta, U. K. Ilyas

**Affiliations:** ^1^ Department of Clinical Pharmacology, Faculty of Medicine, King Abdulaziz University, Jeddah, Saudi Arabia; ^2^ Department of Pharmaceutics, Faculty of Pharmacy, King Abdulaziz University, Jeddah, Saudi Arabia; ^3^ Department of Pharmacognosy and Phytochemistry, Moulana College of Pharmacy, Perinthalmanna, Kerala, India

**Keywords:** *Ocimum sanctum* Linn, galactosamine-induced rat model, hepatoprotective activity, polyphenols, serum biomarkers

## Abstract

*Ocimum sanctum L.* (Tulsi; Family: libiaceae), also known as “The Queen of herbs” or “Holy Basil,” is an omnipresent, multipurpose plant that has been used in folk medicine of many countries as a remedy against several pathological conditions, including anticancer, antidiabetic, cardio-protective, antispasmodic, diaphoretic, and adaptogenic actions. This study aims to assess *O. sanctum* L.’s hepatoprotective potential against galactosamine-induced toxicity, as well as investigate bioactive compounds in each extract and identify serum metabolites. The extraction of *O. sanctum* L as per Ayurveda was simultaneously standardized and quantified for biochemical markers: rutin, ellagic acid, kaempferol, caffeic acid, quercetin, and epicatechin by HPTLC. Hepatotoxicity was induced albino adult rats by intra-peritoneal injection of galactosamine (400 mg/kg). The quantified hydroalcoholic and alcoholic extract of *O. sanctum* L (100 and 200 mg/kg body weight/day) were compared for evaluation of hepatoprotective potential, which were assessed in terms of reduction in histological damage, change in serum enzymes such as AST, ALT, ALP and increase TBARS. Twenty chemical constituents of serum metabolites of *O. sanctum* were identified and characterized based on matching recorded mass spectra by GC-MS with those obtained from the library-Wiley/NIST. We evaluated the hepatoprotective activity of various fractions of hydroalcoholic extracts based on the polarity and investigated the activity at each phase (hexane, chloroform, and ethyl acetate) *in vitro* to determine how they affected the toxicity of CCL4 (40 mM) toward Chang liver cells. The ethyl acetate fraction of the selected plants had a higher hepatoprotective activity than the other fractions, so it was used in vacuum liquid chromatography (VLC). The ethyl acetate fraction contains high amounts of rutin (0.34% w/w), ellagic acid (2.32% w/w), kaempferol (0.017% w/w), caffeic acid (0.005% w/w), quercetin (0.038% w/w), and epicatechin (0.057% w/w) which are responsible for hepatoprotection. In comparison to standard silymarin, isolated bioactive molecules displayed the most significant hepatoprotective activity in Chang liver cells treated to CCl4 toxicity. The significant high hepatoprotection provided by standard silymarin ranged from 77.6% at 100 μg/ml to 83.95% at 200 μg/ml, purified ellagic acid ranged from 70% at 100 μg/ml to 81.33% at 200 μg/ml, purified rutin ranged from 63.4% at 100 μg/ml to 76.34% at 200 μg/ml purified quercetin ranged from 54.33% at 100 μg/ml to 60.64% at 200 μg/ml, purified epicatechin ranged from 53.22% at 100 μg/ml to 65.6% at 200 μg/ml, and purified kaempferol ranged from 52.17% at 100 μg/ml to 60.34% at 200 μg/ml. These findings suggest that the bioactive compounds in *O. sanctum* L. have significant protective effects against galactosamine-induced hepatotoxicity.

## 1 Introduction

Humans relied on medicinal plants for their healing properties centuries before the introduction of chemical medicines. The WHO estimates that around 80% of the people in less developed countries rely exclusively on herbal medicines for their primary healthcare. Medicinal plants serve as the backbone of traditional medicines, and nearly 3.3 billion people regularly utilize plants for therapeutic purposes in less developed countries (Davidson-Hunt, 2000). Medicinal plants are a substantial source of hepatoprotective medications. One estimate places the number of mono- and poly-herbal formulations used for treating different liver problems at over 700 in decoction, tincture, and tablets (Ilyas et al., 2016). The earliest Indian medical system, Ayurveda, has designated numerous plants for the treatment of hepatotoxicity. Given that plants have been employed as medicines for a variety of ailments and with the arrival of current synthetic medicines and their accessibility to consistent dosage forms, usage ease, and therapeutic efficiency in acute circumstances, the usage of medicinal plants has declined (Pandey et al., 2013). Traditional medications have a limited range of action and require long-term administration to be effective, working mostly on chronic illnesses, as opposed to synthetic drugs, which have a restricted spectrum of action and accompanying adverse effects (Fair and Tor, 2014). Numerous plant-based medications have been discovered to have hepatoprotective properties, including *Trigonella foenum graecum* belongs to the family Fabaceae (Zargar, 2014), *Andrographis paniculata* (Family: Acanthaceae) (Nagalekshmi et al., 2011), *Phyllanthus niruri* L. (Family: Phyllanthaceae), *Tephrosia purpurea* L. (Fabaceae), *Boerhavia diffusa* L (Nyctaginaceae) and *A. paniculata* (Family: Acanthaceae) (Dey et al., 2020), *Phyllanthus maderaspatensis* L. (Family: Phyllanthaceae) (Ilyas et al., 2015) and *Fumaria indica* L (Family: Papaveraceae) (Rathi et al., 2008). Jaundice and hepatitis are serious liver diseases with high fatality rates. An infection called hepatitis damages and inflames the liver. Swelling caused by inflammation occurs when bodily tissues are harmed or infected. Hepatitis is often classified as acute or chronic based on how long the liver has been inflamed and damaged (Schaefer and John, 2021). The five fundamental categories of viruses are categories A to E. Due to the weight of illness and mortality, these are of paramount importance. The problem may self-limit (heal on its own) or it may worsen, leading to cirrhosis and fibrosis (Eming et al., 2017). Aside from alcohol, drugs, chlorinated solvents, herbal remedies, chlorinated solvents, peroxidized fatty acids, industrial pollutants, radioactive isotope intoxication, and fungal toxins, other xenobiotics that can cause hepatic problems include parasite and viral infections, autoimmune diseases, and radio-active isotopes. In particular, types A and C induce chronic illness and are the main factors in liver cirrhosis and cancer, respectively (Ilyas et al., 2016). Only a few uncommon allopathic hepatoprotective medications are currently available for the treatment of liver disorders. Plant extracts are therefore frequently used to treat liver problems. India’s semi-tropical and tropical regions are home to *Ocimum sanctum* Linn. Ayurveda and Siddha traditions have historically used various components of the plant to treat a variety of illnesses, including infections, skin problems, and hepatic abnormalities, and as an antidote for snake and scorpion stings. This plant’s leaves have long been utilized for their anti-inflammatory, gastroprotective, and hepatoprotective effects (Kamyab and Eshraghian, 2013), anti-plasmodial, neuroprotective, and chemo-preventive properties (Bhattacharyya and Bishayee, 2013; Rajendran et al., 2014; Mataram et al., 2021). The plant is also effective against human pancreatic cancer, stress-induced anxiety, stress-induced oxidative and central monoaminergic changes, typhoid fever, and cerebral ischemia/reperfusion (Ahmad et al., 2012a; Ahmad et al., 2012b; Mandal et al., 2012; Shimizu et al., 2013; Singh et al., 2016). In COVID-19, Tulasi has been found to possess SARS-CoV-2 protease inhibition, lipid-lowering and antioxidant, anxiety and depression (Chatterjee et al., 2011; Suanarunsawat et al., 2011; Shree et al., 2020). This plant also contains a high concentration of polyphenolic chemicals, which have a variety of biological activities such as antiviral, antibacterial, vasodilatory, antioxidant, anti-inflammatory, and antiradical properties (Prochazkova et al., 2011), memory improvement (Spencer, 2009), gastroprotection (Mota et al., 2009), antioxidation, anti-inflammation, cardiovascular protection (Garcia-Lafuente et al., 2009), chemopreventive activity (Mohan et al., 2013) and regulate immune responses (Magrone et al., 2008). Previous research found that these plants possessed a high concentration of flavonoids, which are naturally occurring phenolic phytochemicals that have been linked to a variety of biological functions (Hollman and Katan, 1999), flavonoid glycoside is as a multi potent bioflavonoid with great potential for the prevention and treatment of disease, kaempferol glycosides is antinociceptive and anti-inflammatory (De Melo et al., 2009), anti-tyrosinase activity (Rho et al., 2010). Previously, metabolic profiling was used for a better understanding of the chemical diversity of medicinal plants. This information can be used to make comparisons with other studied taxonomically related plants and to infer their bioactivity. Chromatography coupled with mass spectrometry is the most widely used technology for analyzing samples in extremely complex matrices, such as plant extracts (Jorge et al., 2016). Both morphologically and functionally, the galactosamine-induced experimental model system in rats is known to resemble viral hepatitis in people. Because hepatocytes have significant quantities of galactokinase and galactose-1-uridylyltransferase, galactos-amine has a greater liver selectivity than other hazardous groups, such as acetaminophen, paracetamol, and carbon tetrachloride. Hepatotoxicity, substantial portal and parenchymal infiltration, and patchy hepatocyte necrosis are all induced by galactosamine (Ilyas et al., 2015). By boosting the creation of UDP-sugar derivatives, galactosamine also causes the depletion of uridine diphosphate (UDP), which inhibits the synthesis of RNA and proteins and, in turn, deteriorates cell membranes. Chattopadhyay et al. (1992) and Jain (2015) revealed that only crude extracts are responsible for activity but did not isolate active ingredients. We identified, evaluated and isolated the most active bioactive molecules responsible for hepatoprotection in this study. The plan of the study was focused on bioactivity guided fractionation, *O. sanctum* (ethyl acetate) fractions showed significant hepatoprotective activity (cell induced with carbon tetrachloride 40 mM) as compared with other fractions, which led to the further separation of ethyl acetate fraction by vacuum Liquid chromatography and followed by the quantitative chemo profiling of the potent fraction. The chemical constituents were potent activity responsible for hepatoprotective activity will be identified and validated. A single newly developed solvent system formic acid, ethyl acetate, and toluene (1:4:5) was used for the densitometric quantification of bioactive compounds by HPTLC in aqueous alcoholic extracts with reference to respective marker compounds such as ellagic acid, rutin, kaempferol, quercetin, caffeic acid, and epicatechin in *O. sanctum.* The purpose of this study was to use GC-MS in order to investigate the metabolic profile of the hexane of the serum metabolites of *O. sanctum* and to investigate potent bioactive compounds obtained from *O. sanctum* for their hepatoprotective activity.

## 2 Material and methods

### 2.1 Reagents and chemicals

We bought reference standards from Natural Remedies Pvt. Ltd., including rutin, kaempferol, quercetin, epicatechin, caffeic acid, and catechin (Bangalore, India). We bought ALT, AST, and ALP kits from Span Diagnostics Ltd. in Surat, India. The supplier of galactosamine was SRL in Mumbai, India. Analytical-grade chemicals were employed throughout. As mobile phases for a HPTLC analysis, formic acid, ethyl acetate, toluene, and methanol (CDH Labs, Mumbai, India) were utilized. A 0.22 μm syringe-driven filter was utilized to filter all the solutions for the analysis (HIMEDIA, Mumbai, India).

### 2.2 Extract preparation

Fresh plant material was taken from the Maruthmallai region of Kanyakumari district, Tamilnadu, India. It was identified and authenticated by Dr. V. Chelladurai, Research Officer, Central Council for Research in Ayurveda and Siddha (Govt. of India), Tirunelveli, Tamil Nadu, India. A voucher specimen has been stored in our laboratory for future reference. Plant materials were extracted using 95% alcohol for 6 h at 37°C and an aqueous-alcoholic solvent (50%) for 5 h at 38°C. The extraction process was carried out three times. The mixed extracts were collected and dried in a rotary evaporator at 40°C under decreased pressure.

### 2.3 Animals experimental design

The Central Animal House facility (Registration No. 173/CPLSEA/837) provided adult Wistar rats weighing 150–200 g. The animals were fed a typical rodent diet and given access to water as needed while being kept in regular laboratory conditions (12 h light/dark cycles at 25°C with humidity levels between 45% and 65%). The animals were divided into eight groups (*n* = 6) at random. Group I acted as the vehicle control and was given regular saline for a period of 7 days. In addition to receiving normal saline (1 ml/kg, p.o.) for 7 days, Group II acted as the toxic control group. Groups III to VIII received a prophylactic treatment of a hydroalcoholic and alcoholic extract of *O. sanctum* in carbon methyl cellulose (0.1%) at various concentrations (100 and 200 mg/kg b.w.p.o) for 7 days. Silymarin (40 mg/kg, p.o.) was administered to Group VIII for 7 days. The current study evaluated the dose of *O. sanctum* extracts based on prior research studies conducted by Chattopadhyay et al. (1992). On the eighth day, 400 mg/kg ip of galactosamine was administered to Groups II to VIII to cause liver damage (Raish et al., 2016).

#### 2.3.1 Evaluation of liver function

After giving galactosamine for 24 h, blood was drawn from the retro-orbital plexus while the groups were lightly sedated with ether (Van Herck et al., 2001). All of the groups were sacrificed immediately following blood collection. Samples of the liver were obtained for histopathological and biochemical analyses. At 37°C, serum was centrifuged to separate it, and it was then used to measure various biochemical characteristics. Using a motor-driven Teflon pestle, 10% (w/v) liver homogenates were produced in an ice-cold 0.15 M KCl solution after the liver samples were rinsed with chilled normal saline and weighed (AlSaid et al., 2015). Additional biochemical parameters, including the determination of aspartate amino transaminase (AST), alanine amino transaminase (ALT), and alkaline phosphatase (ALP), were estimated using the serum (Patlolla et al., 2011). The liver homogenate’s supernatant was used to measure antioxidant enzymes, such as catalase (CAT) and superoxide dismutase (SOD), using a colorimetric technique (Weydert and Cullen, 2010). The DTNB method was used to calculate glutathione (GSDH), and a modified approach was used to calculate TBARS, an index of lipid peroxidation (Liu et al., 2020). The quickly excised liver tissues were stored in neutral buffered formalin. According to Badawi’s instructions, liver slices were created for histological investigations (Badawi, 2019).

### 2.4 GC-MS analysis of metabolic serum

An analysis was performed on the chemical makeup of the metabolic serum. Gas chromatography (GC-MS) was used to characterize 20 active compounds. The Agilent 7890AGC system was connected to a 5975C inert XL EI/CI MSD mass spectroscopic system, which was outfitted with a 30 m × 250 μm × 0.25 µm HP-5MS capillary column. A CTCCombiPAL injector was used, and the injection volume was 2.0 L. With a helium flow rate of 1.0 ml·min^−1^, the inlet temperature was maintained at 270°C. Programmatically, the column’s temperature was increased from 60°C to 300°C at a rate of 5°C per minute for a total run period of 40 min in SCAN mode.

#### 2.4.1 Preparation of sample

The serum of the *O. sanctum* extract (5 ml) was extracted with acetonitrile (3 × 100). The combined acetonitrile layer was evaporated to dryness under reduced pressure at 40°C in a rotary evaporator to obtain a residue. The residue was dissolved in LC-MS-grade methanol, filtered through a 0.22 µm syringe, and injected into GC-MS.

### 2.5 Densitometric quantification of bioactive compounds in ethyl acetate fraction using HPTLC

#### 2.5.1 Sample preparations

The aqueous ethanolic extract of *O. sanctum* was dissolved in 10% distilled water and was successively fractionated thrice with hexane (3 × 600 mL), chloroform (3 × 600 mL), ethyl acetate (3 × 500 mL), and water-soluble fractions. The combined fractions of *O. sanctum* were evaporated to dryness under reduced pressure at 40°C in a rotary evaporator. 100 mg of ethyl acetate fraction were accurately weighed, dissolved in 10 mL of HPLC grade methanol, sonicated for 10 min, and then made up with 10 mL of methanol. After filtering the solution, a 0.22 µm syringe was used to inject it into the HPTLC system.

#### 2.5.2 Preparation of standard solutions

The standard solution was made by dissolving precisely weighed 10.0 mg of rutin, kaempferol, quercetin, ellagic acid, and epicatechin in 10.0 mL of methanol HPLC grade as stock solution and storing it at 4°C. These standards were further diluted to achieve the desired concentration for quantification.

#### 2.5.3 Preparation of the plate

Prior to usage, pre-coated silica gel 60 F254 aluminium HPTLC plates (Merck, Germany) were washed in methanol and dried. The standard solution of bioactive compounds and samples were applied on an HPTLC plate in the form of bands of 4 mm width using a Linomat V applicator (Muttenz, Switzerland) with a 100 µL syringe. The application rate was kept constant at 200 nL·s^−1^, and the space between the two bands was 9 mm.

#### 2.5.4 Calibration curve of bioactive compounds

Standard concentrations of rutin (10–1,600 ng/band), epicatechin (100–5,000 ng/band), ellagic acid (20–200 ng/band), kaempferol (40–200 ng/band), quercetin (10–160 ng/band) were applied in triplicate on silica-gel 60 F254 plates using a CAMAG Linomat-5 Automatic Sample Spotter. The plates were developed in formic acid, ethyl acetate, and toluene (1:4:5 v/v/v) solvent in a CAMAG glass twin-trough chamber (20 cm × 100 cm) up to a distance of 8 cm. After development, the plates were dried in air and scanned at 366 nm using a CAMAG TLC Scanner 3 and Win CATS 4 software. The peak areas were recorded. Calibration curves for bioactive compounds were created by plotting peak areas versus applied ethyl acetate fractions conaining rutin, epicatechin, ellagic acid, kaempferol, quercetin respectively.

### 2.6 Separation and purification of bioactive compounds

The ethyl acetate fraction has been shown to have a strong hepatoprotective impact in comparison to other fractions. To separate the various components contained in the fraction, vacuum liquid chromatography (VLC) was used on this fraction. Dry slurry was made by mixing a small amount of Silica gel G (Merck) with 15 g of ethyl acetate, and this slurry was then placed onto a sintered glass funnel with Silica gel G as the stationary phase. The column was eluted with solvents of increasing polarity step-by-step under a vacuum, starting with a blend of pure toluene and ethyl acetate and finishing with pure ethyl acetate. To further clarify, the toluene levels were decreased when the ethyl acetate component was increased after initially eluting with 5% ethyl acetate in toluene. The ethyl acetate component was then increased by 5% increments up to 50% and then by 10% increments up to 100% ethyl acetate. The methanol content was increased in 5% steps after elution with ethyl acetate, elution with 5% methanol in ethyl acetate, and elution with 100% methanol. The solvents were eluted until they ran clear from the funnel. Throughout the experiment, a steady flow rate of the solvent (100 ml/min) was observed. Individual fractions were collected, and their homogeneity was checked using TLC. The same R_f_ values from similar fractions were merged and crystallized.

### 2.7 HPTLC study of column eluents of ethyl acetate fraction

The fractions obtained from the VLC were analyzed by using HPTLC, and fractions with a similar profile were combined. Aliquots of these fractions were reconstituted in methanol and subjected to an HPTLC study. Small quantities of dried eluents were dissolved in about 1 ml of HPLC-grade methanol. The sample was sprayed onto a plate at a distance of 0.8 cm from one end of the plate in the form of a narrow band using the spray-on technique with the help of a Linomate-V applicator attached to a CAMAG HPTLC system that was programmed using win-CATS software, which was included with the apparatus. Following the application of the spot, a chromatogram was generated using several solvent systems in a twin-trough chamber (20 cm × 25 cm) that had previously been saturated with a formic acid, ethyl acetate, and toluene combination (1:4:5 v/v/v). The air-dried plate was viewed under the different wavelengths of UV light and visible light. The spots were scanned using Reprostar 3; the TLC scanner 3 was designed for the densitometric evaluation of plates.

### 2.8 Hepatoprotective assay of fractions, bioactive compounds, and column eluents of ethyl acetate fraction

Cells were plated in 96-well plates at a density of 1 × 10 ^6^ cells/well and allowed to grow there overnight. After 24 h, the medium was removed, the cells were given different quantities of treatment of each sample (100 and 200 μg/ml), and they were then incubated for 2 h in separate wells of a 96-well plate. As a benchmark, silymarin (100 and 200 μg/ml) was used. CCl4 was applied to the cell and incubated with it for 2 h. Following incubation, the cells were washed, and each well received 20 μL of MTT (5 mg/ml of MTT in PBS) for 1 h. After 2 h, a microscope was used to witness the formation of a formazan crystal. An additional hour of incubation was added if the crystal formation was not accurate. Following the removal of the medium, the leftover formazan crystals in each well were dissolved in 200 μL of DMSO. The cell culture plate was shaken for 15 min, while the absorbance was measured at 540 nm using an ELISA reader.

The following formula was used to calculate the percentage of the hepatoprotection of the polyphenols, fractions, column eluents, and silymarin:

$$\text{Percentage Hepatoprotection}=\left(\text{OD of Test sample }/\text{ OD of Control}\right)\;X\;100$$ (1)

### 2.9 Statistic evaluation

The results are given as mean S.E.M. Dunnett’s *post hoc* test was used after a one-way analysis of variance (ANOVA) to estimate the total variation present in a data set. *p* < 0.01 was regarded as statistically significant.

## 3 Results and discussion

### 3.1 Effect of different extracts of *O. sanctum* on biochemical parameters in rats

**FIGURE 1:**
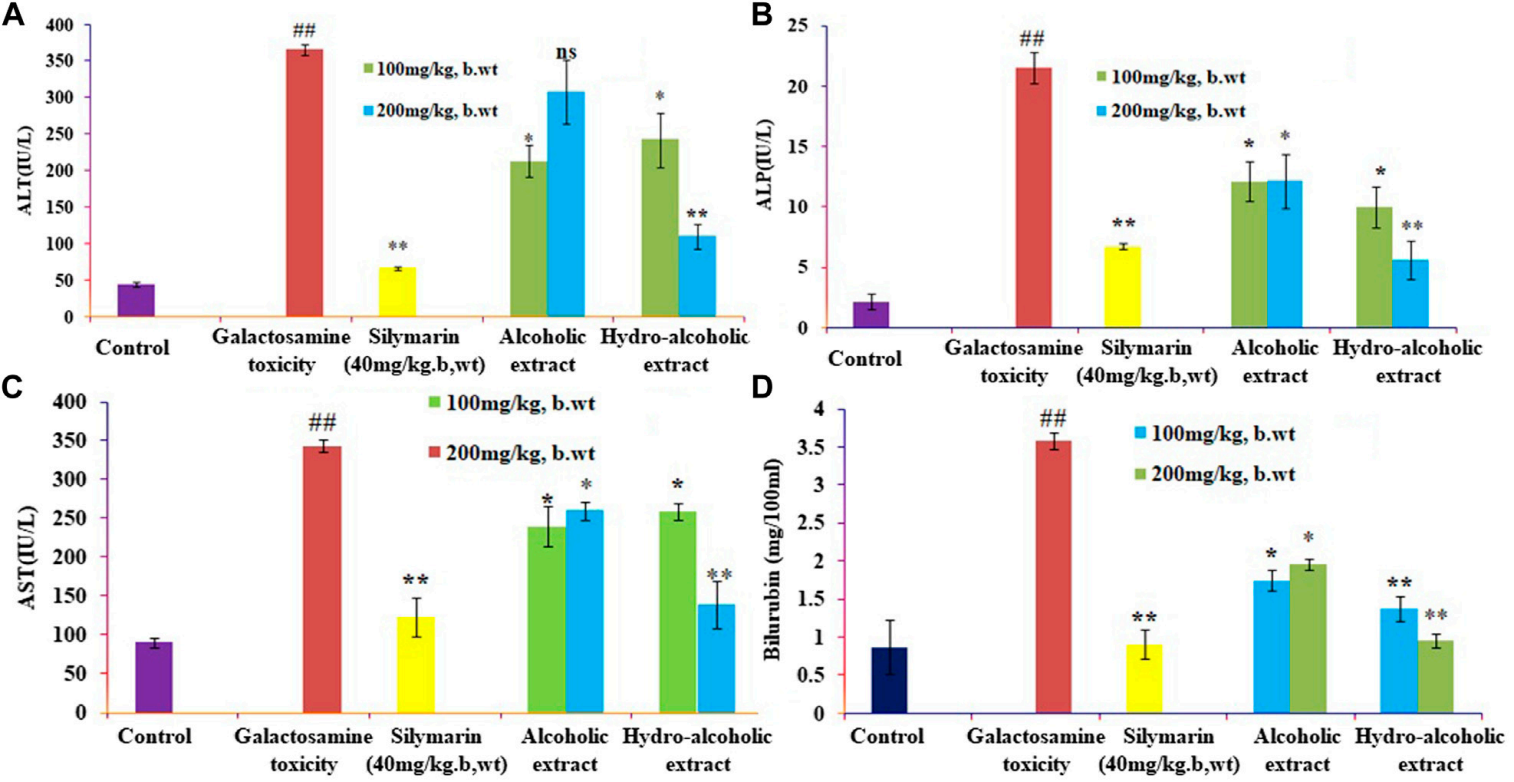
Effects of alcoholic and hydroalcoholic extracts (at two different doses of 100 and 200 mg/kg body weight, administered orally for 7 days) and standard silymarin on biochemical parameters such as ALT or alanine aminotransferase **(A)**, ALP or Alkaline phosphatase **(B)**, ASTor aspartate aminotransferase **(C)**, and bilirubin **(D)** of liver on oxidative stress induced by galactosamine. GalN was administered intraperitoneally on the eighth day as a pretreatment. Each value represents means ± standard error of the mean. ns *p* > 0.05 (non-significant), *p < 0.05 (significant), **p < 0.01 (more significant).

**FIGURE 2:**
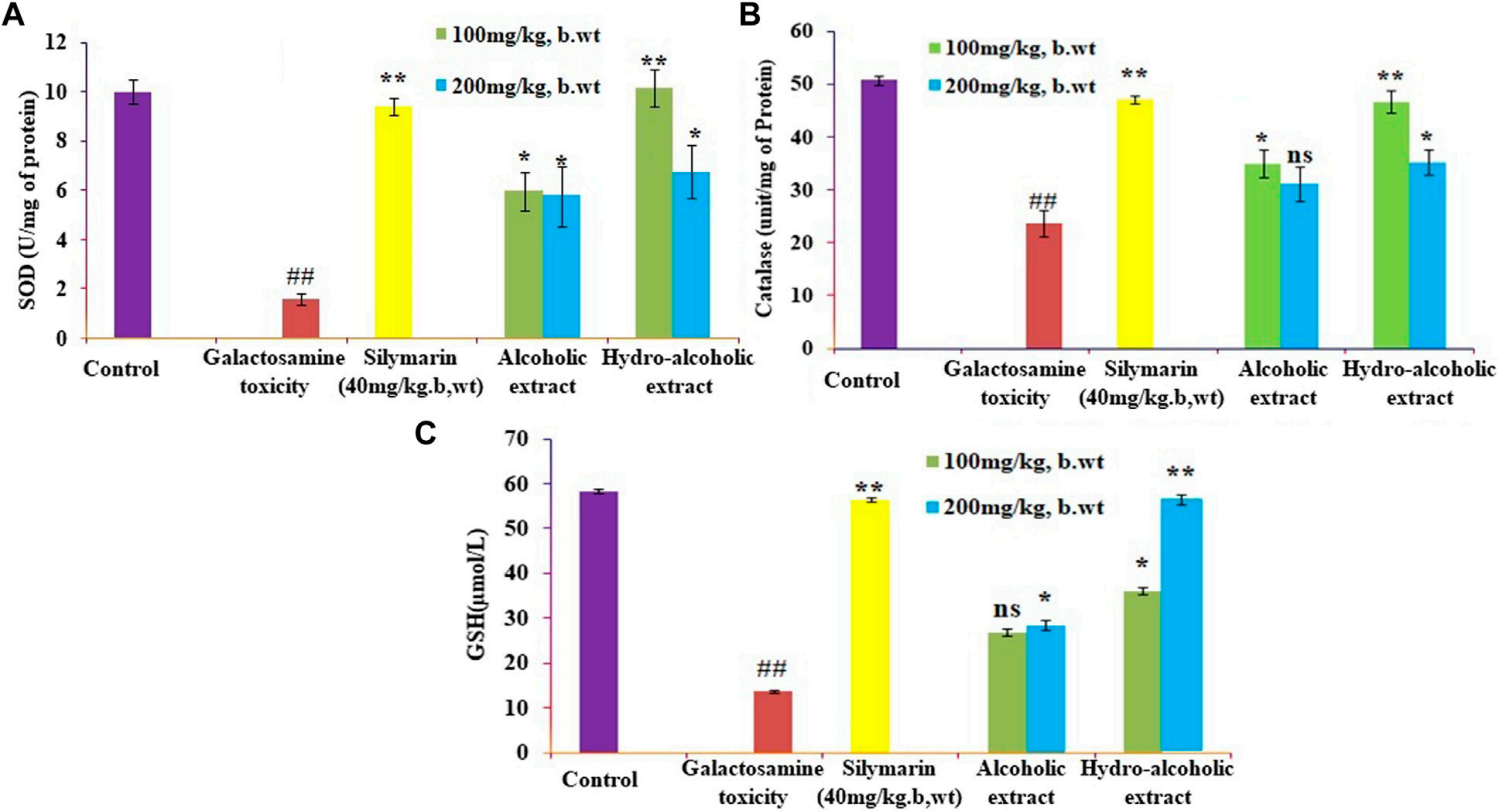
Effects of alcoholic and hydroalcoholic extracts (at two different doses of 100 and 200 mg/kg body weight, administered orally for 7 days) and standard silymarin on antioxidant parameters such as SOD or Superoxide dismutase **(A)**, GSH or Glutathione **(B)**, and CAT or catalase **(C)** of the liver on oxidative stress induced by galactosamine. GalN was administered intraperitoneally on the eighth day as a pretreatment. Each value represents means ± standard error of the mean. ns *p* > 0.05 (non-significant), *p < 0.05 (significant), **p < 0.01 (more significant).

The present study showed that analyzed biochemical markers, which included AST, ALT, ALP, and TBARS Figures 1A–D respectively, were found to be considerably higher in galactosamine-treated rat compared to the normal control group (*p* < 0.01) There was also a substantial drop in SOD, CAT, and glutathione Figures 2A–C respectively levels in the tissue (*p* > 0.05). Hydroalcoholic extract was found to be more effective than ethanolic extracts. Figures 1, 2 shows the effect of alcoholic, hydroalcoholic and silymarin pretreatment on biochemical parameters of galactosamine-intoxicated rats. Previous study, only *O. sanctum* extract has been reported to have hepatoprotective activity against paracetamol-induced liver damage in Albino rats (Lahon and Das, 2011). The hepatoprotective and antioxidant activities of the crude fractions of the endophytic fungi of *O. sanctum* in rats were reported by Shukla et al. (2012) revealed that only crude extracts are responsible for activity but did not isolate active ingredients. Present investigation, the hepatoprotection may be attributable to numerous bioactive active moieties discovered in high concentrations in the hydroalcoholic extract of *O. sanctum*. Such moieties include bioactive compounds were simultaneously quantified using HPTLC. Bioactive active moieties act as potent free radical scavenging ability of hydroalcoholic extract provides evidence for the role of tannins and flavonoids as potential secondary metabolite responsible for hepatoprotection (Li et al., 2018). Their action may be mediated by preventing liver injury by stopping the development of lipid peroxides and inhibiting oxidative mechanisms that contribute to hepatocyte degeneration (Cichoż-Lach and Michalak, 2014).

### 3.2 Histopathological observation

**FIGURE 3:**
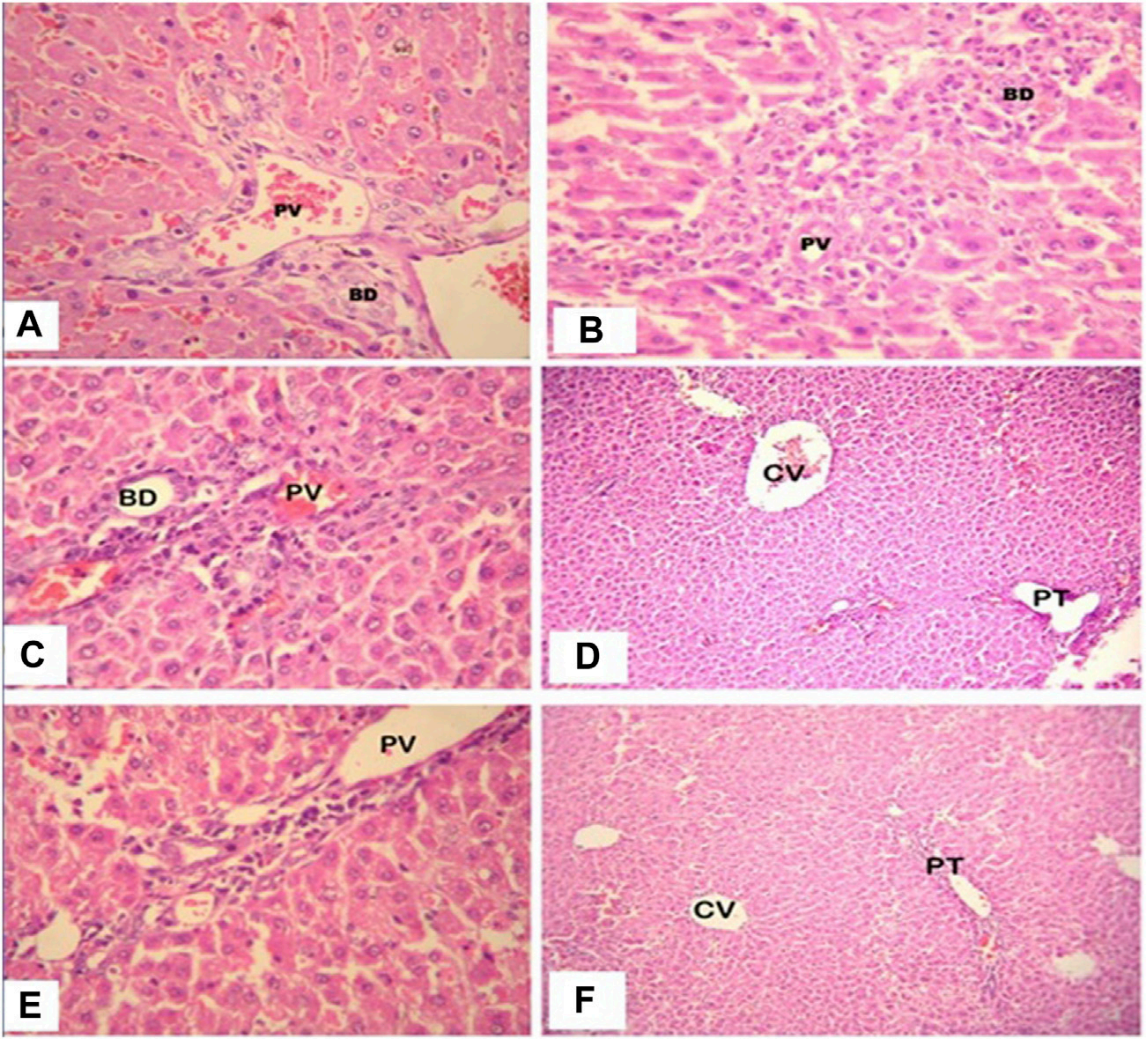
High power photomicrograph (400x) of liver section, the control group **(A)** shows normal hepatic architecture; in the galactosamine-induced necrotic liver cells and focal hemorrhages **(B)**; in the alcoholic extracts plus galactosamine **(C,D)**, there is moderate inflammatory cell infiltration; and in the hydroalcoholic extracts plus galactosamine **(E,F)**, there is only mild inflammatory cell infiltration.

Following histology, the normal control animals’ livers had a well-defined central vein, a well-preserved cytoplasm, and a conspicuous nucleus and nucleolus. The galactosamine-treated animals’ liver sections revealed severely toxic liver cells that had necrotic liver cells, focal hemorrhages in the periportal region, inflammatory cell collection, and distributed inflammation throughout the liver parenchyma. The hepatic cells with a well-preserved cytoplasm revealed that the *O. sanctum* hydroalcoholic extracts appeared to be more significant than the other extracts for the avoidance of galactosamine toxicity. Silymarin also defended against the hepatic alterations induced by galactosamine. Further, the hepatotoxic galactosamine-treated rats’ liver sections underwent a histological analysis, which revealed a clear inflammatory cell infiltration around the portal triad. In the periportal region, it was found that there were necrotic liver cells and localized bleeding. However, the absence of necrosis and the development of normal hepatic cells in the liver sections of the rats given silymarin and the alcoholic and hydroalcoholic extracts of *O. sanctum* in various groups indicated signals of protection at a significant level. Figure 3 shows a histopathologic segment of liver (400X) stained with hematoxylin and eosin to reveal the cell nucleus and cytoplasm with extracellular matrix in purple blue and pink colours, respectively.

### 3.3 Metabolic fingerprinting of faded extract serum by GC-MS

The immunomodulatory activity of crude extract in the previous investigation by Bochu et al. (2005) was not significantly affected; however, crude extract metabolites exhibited higher immunomodulatory action than crude extract. In the current investigation, a thorough analysis was done with reference to Bochuet et al. Rat feeding trials were done with various quantities of aqueous-alcoholic (50%) extracts of chosen medicinal plants (100 and 200 mg/kg, b/w). The serum metabolites were initially analyzed using HPTLC. No distinct separation/peak was observed, which indicated that the serum was a mixture of compounds. Subsequently, the serum was analyzed using GC-MS. The serum metabolites from the extract-fed rats were identified using GC-MS, which were found to be fatty and aliphatic compounds. The process of identifying bioactive components was based on comparing the recorded mass spectra to those received from the Wiley/NIST collection. Metabolite serum, which is described in Table 1, characterized 20 different components in total. A literature review revealed that the discovered serum metabolites had no significant impact on hepatoprotective activity, thus we concentrated primarily on the ethyl acetate fraction, which contained the most significant bioactive molecule for hepatoprotection.

**TABLE 1:** Identification of serum metabolites of crude extract by GC-MS.

	R.T.	Area	Compound name		R.T.	Area	Compound name
1	11.733	0.65	1-tetradecene	21	25.176	1.83	Linoleic-9-Octadecyne
2	14.397	7.16	2,4-Di-tert-butylphenol	22	25.255	3.47	trans-13-Octadecenoic acid
3	14.681	0.86	Benzoic acid, 4-ethoxy-, ethyl ester	23	25.587	2.65	Octadecanoic acid
4	15.854	3.01	1-Hexadecene	24	25.926	0.72	Docosyl acetate
5	18.379	0.45	Methyl tetradecanoate	25	26.953	1.31	Methyl arachidonate
6	19.116	1.54	Tetradecanoic acid	26	27.617	0.41	2-Monoolein
7	19.545	3.08	1-Octadecene	27	29.079	0.5	N-Allylphthalimide
8	20.965	0.83	Butyl octyl phthalate	28	29.394	0.27	Cis-4-methyl-exo-tricyclo[5.2.1.0(2.6)]decane
9	21.744	0.52	Dibutyl phthalate	29	30.33	2.06	1-Monopalmitin
10	21.835	6.17	Hexadecanoic acid	30	32.904	1.1	1-Monostearin
11	21.938	0.34	2-Hexyl-3,5-dinotrobenzonitrile	31	33.2	7.66	1-Benzylidene-5,6-butano-7-azaindane
12	22.198	1.53	3,8-Di-tert-butyl-1,10-phenanthroline	32	33.6	1.15	16,17-bis(trimethylsilyloxy)androsta-1,4-diene-3-methyloxime
13	22.53	15.56	Elaol	33	34.1	0.79	Phthalic acid, isopropyl pentyl ester
14	22.729	0.3	l-(+)-Ascorbic acid 2,6-dihexadecanoate	34	34.2	0.44	Bis(7-methyloctyl) phthalate
15	22.88	2.59	Tetradecyl heptafluorobutyrate	35	34.4	0.57	Phthalic acid, butyl isopropyl ester
16	24.028	0.6	Butyl phthalate	36	34.7	0.37	Phthalic acid, neopentyl 2-propyl ester
17	24.518	1.73	Linoleic acid methyl ester	37	36.5	0.73	Propenocarbachlorin
18	24.602	2.95	Elaidic acid methyl ester	38	37.5	16.7	Cholest-5-en-3-ol (3.beta.)
19	24.693	0.76	Methyl elaidate	39	38.0	0.92	Perhydro-htx-2-one, 2-depentylacetate ester
20	24.983	3.24	Methyl stearate				

### 3.4 HPTLC quantification of bioactive compounds in ethyl acetate fraction

#### 3.4.1 Development of the perfect mobile phase

**FIGURE 4:**
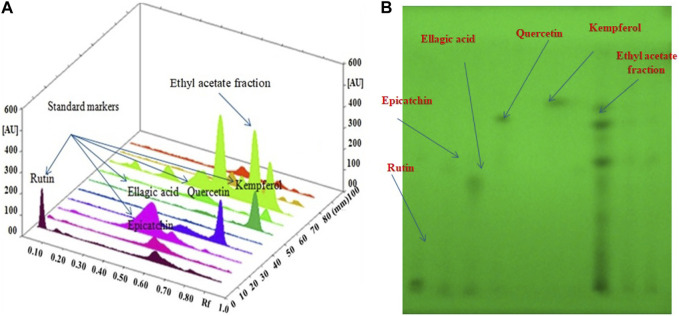
**(A)**: 3D image of all tracks at 254 nm demonstrating that the ethyl acetate fraction had bioactive compound; **(B)**: HPTLC Chromatogram of the ethyl acetate fraction contained bioactive compound at 254 nm in the solvent system: a combination mixture of formic acid: ethyl acetate:toluene (1:4:5 v/v/v).

For the simultaneous estimation of bioactive compounds, different proportions of toluene, ethyl acetate such as toluene/ethyl acetate (3:0 v/v), toluene/ethyl acetate (5:5 v/v), toluene/ethyl acetate (7:3 v/v), toluene/ethyl acetate/methanol (7:2.8:0.2 v/v/v), toluene/ethyl acetate/formic acid (7:2.5:0.5 v/v/v) and toluene/ethyl acetate/formic acid (5:4:1 v/v/v) were evaluated as the solvent systems for the development of a suitable band. All investigated solvent systems were developed under chamber saturation conditions. From the obtained results, it was observed that the solvent systems toluene/ethyl acetate (3:7 v/v), toluene/ethyl acetate (5:5 v/v), toluene/ethyl acetate (7:3 v/v), toluene/ethyl acetate/methanol (7:2.8:0.2 v/v/v), toluene/ethyl acetate/formic acid (7:2.5:0.5 v/v/v) offered the poor densitometry peaks of bioactive compounds with tailing factor. However, when the toluene/ethyl acetate/formic acid (5:4:1v/v/v) was studied, it was observed that this solvent system offered a well-separated and intact chromatographic peak of rutin at R_f_ = 0.08, ellagic acid at R_f_ = 0.55, quercetin at R_f_ = 0.62, kaempferol at R_f_ = 0.67 and epicatechin at R_f_ = 0.57 respectively. The typical band of the bioactive compounds and ethyl acetate fraction of *O. sanctum* was scanned at 254 nm (Figure 4). Vasilisa et al. (2020) also established the mobile phase mixture of diethyl ether, formic acid, acetic acid, water, acetophenone and heptane (30:3:9:50:30:10) (v/v/v/v/v/v) (Pedan et al., 2020). Ilyas et al. (2015) reported a solvent mixture of toluene: ethyl acetate, formic acid and methanol (3:3:0.8:0.2) (v/v/v/v) respectively, for simultaneous quantification of bioactive compounds (Ilyas et al., 2015). Bioactive compounds are naturally occurring substances with the potential to protect the liver from damage and act as antioxidants (Kamyab and Eshraghian, 2013; Saha et al., 2019). The hepatoprotective and antioxidant potential of bioactive compounds may be due to their capability to normalize impaired membrane function (De Oliveira e Silva et al., 2012).

### 3.5 Hepatoprotective activity of fractionated extracts of *O. sanctum*

**FIGURE 5:**
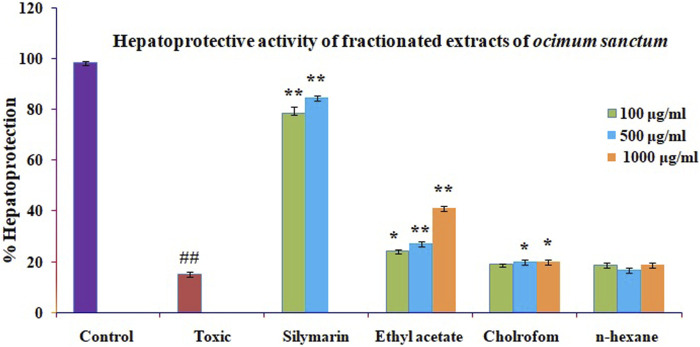
Effect of different fractions on toxicity caused by GlaN in Chang liver cells. Before being treated with GalN (40 mM) for 2 h, Chang liver cells were first incubated for 2 h in the presence or absence of extracts and their metabolites. The cells were then prepared for the MTT experiment. The findings are presented as mean S.E.M. Compared to the GalN (40 mM) control, ns *p* > 0.05, **p < 0.01, and *p < 0.05 are significant.

Harsha et al. (2020) investigated cytotoxicity assay of *O. sanctum* extract on leukemic cell lines: A preliminary *in-vitro* study. We are looking into the cytotoxicity of extracts against Chang liver cells. Drugs concentrations ranging from 100 to 1000 μg/ml were used to evaluate the percentage growth inhibition of the drugs on cell lines. The drug sample exhibited a CTC50 value greater than 1000 μg/ml (concentration required to inhibit viability by 50%). We assessed the hepatoprotective activity of several *O. sanctum* fractions based on polarity and studied the activity in each phase (hexane, chloroform, and ethyl acetate) as well as against CCl4-induced hepatotoxicity. The CCL4 treatment of Chang liver cells resulted in a considerable drop in cell viability. When compared to the other fractions, treatment with the ethyl acetate soluble fractions showed more significant dose-dependent protection against cell damage brought on by CCL4 exposure. The results are presented using the formula mean standard error mean (S.E.M.). Figure 5 shows the percentage of protection graphically.

### 3.6 Vacuum liquid chromatography of potent ethyl acetate fraction

The ethyl acetate fraction of *O. sanctum* was used to perform vacuum liquid chromatography, yielding twenty-eight column eluents. HPTLC was used to evaluate these column eluents in order to identify bioactive compound from the ethyl acetate fraction of *O. sanctum* (Table 2). The different components contained in the eluents were detected by spraying with NP reagents and then comparing the Rf value to the standards (Gallic acid, ellagic acid, Kaemferol, quercetin, rutin, epicatechin, ursolic acid and catechin). These bioactive compounds are responsible for a variety of biological functions, including anticancer (Horcajada-Molteni et al., 2000), anti-oxidant (Afanas’ev et al., 1995), chemopreventive (Araujo et al., 2011), and immunosuppressive (Yu et al., 2011). We selected the most powerful bioactive molecule for hepatoprotection in the current investigation and subsequently isolated active moieties.

**TABLE 2:** Solvent combinations and R_f_ value of column eluents of ethyl acetate fraction of *O. sanctum.*

Tracks	Solvent combination	R_f_	Tracks	Solvent combination	R_f_
01	Gallic acid	**0.52**	20	Toluene + 25% ethyl acetate-1	**0.62**
02	Ellagic acid	**0.55**	21	Toluene + 25% ethyl acetate-2	0.52, 0.63, 0.74
03	Kaempferol	**0.81**	22	Toluene + 25% ethyl acetate-3	0.54
04	Quercetin	**0.73**	23	Toluene + 50% ethyl acetate-1	0.34, 0.43, 0.58, 0.77
05	Rutin	**0.08**	24	Toluene + 50% ethyl acetate-2	0.31, 0.45, 0.54,0.63
06	Epicatechin	**0.50**	25	Toluene + 50% ethyl acetate-3	0.31, 9.37, 0.45, 0.48, 0.54, 0.57, 0.62, 0.68, 0.73
07	Catechin	**0.54**	26	Toluene + 50% ethyl acetate-4	0.31, 0.38, 0.43, 0.57, 0.71
09	Caffeic acid	**0.63**	28	Toluene + 75% ethyl acetate-1	0.09, 0.32, **0.50**, **0.57**
10	Ursolic acid	Nil	29	Toluene + 75% ethyl acetate-2	0.81
11	100%Toluene-1	0.81	30	Toluene + 75% ethyl acetate-3	0.1, 0.18, **0.23**, 0.28, 0.38, 0.55
12	100% Toluene-2	0.76	31	100% ethyl acetate	0.08, **0.53**
13	10%Toluene-	**0.67**	32	5%methanol + ethyl acetate	—
14	10%Toluene-2	0.68, 0.76	33	15%methanol + ethyl acetate	—
15	Toluene + 10% ethyl acetate	0.77, 0.61	34	25% methanol + ethyl acetate-1	0.10, 0.30, 0.48, **0.52**, 0.61
16	Toluene + 10% ethyl acetate	0.61, 0.69, 0.78	35	25%methanol + ethyl acetate-2	0.06
17	Toluene + 10% ethyl acetate	0.73	36	25%methanol + ethyl acetate-3	0.32, 0.63
18	Toluene + 10% ethyl acetate	0.73	37	25%methanol + ethyl acetate-4	—
19	Toluene + 20% ethyl acetate	0.73, 0.81	38	50%methanol + ethyl acetate-	**0.63**

The following were used to identify the standards (1–10): Track No. 31 (100 percent ethyl acetate elute) was connected to ellagic acid (RF, value: 0.53); Track No. 13 (10% ethyl acetate in toluene) was connected to kaempferol (RF, value: 0.67); Track No. 20 (25% ethyl acetate in toluene elute) and quercetin (Rf value: 0.62) were identified; Track No. 28 (75% ethyl acetate in toluene elute) was paired with epicatechin (RF, value: 0.57); and rutin corresponded to Track No. 5 (Toluene + 50% ethyl acetate-2, elute), with an Rf value of 0.08.

Rf value was shown by bold values. Ethyl acetate fraction contained active substances and standard biomarkers were matched.

### 3.7 Hepatoprotective activity of column eluents of ethyl acetate fractions

**FIGURE 6:**
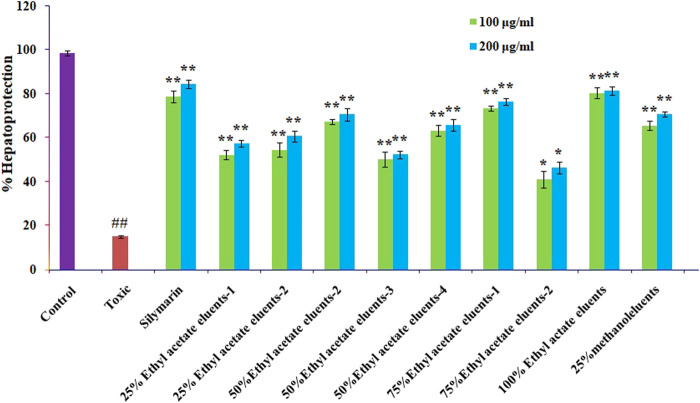
Effect of different columns eluting *O. sanctum*’*s* ethyl acetate on CCl4-induced toxicity in Chang liver cells. Prior to being treated with CCl4 for 2 h, Chang liver cells were cultured for 2 h in the presence or absence of various columns elutes of *O. sanctum*’*s* ethyl acetate. The cells were then prepared for the MTT experiment. The findings are presented as mean S.E.M. Compared to the CCL4 control, ns *p* > 0.05, **p < 0.01, and *p < 0.05 are significant.

The hepatoprotective activity of the ten ethyl acetate column eluents previously mentioned against CCL4-induced cytotoxicity was examined by pre-incubating the cells with or without the ethyl acetate fractions or silymarin. The Chang liver cells exposed to CCl4 saw a significant reduction in cell viability. The mean standard error means (S.E.M.) formula was used to present the results. The proportion of protection is graphically shown in Figure 6. The elute from the ethyl acetate (100%) column showed that ellagic acid has significant hepatoprotective effects, followed by 75% ethyl acetate elutes (catechin and epicatechin), 50% ethyl acetate (rutin), 25% ethyl acetate (quercetin and kaempferol), and 25% methanol (caffeic acid), when compared with standard silymarin.

### 3.8 Hepatoprotective activity of isolated polyphenols

**FIGURE 7:**
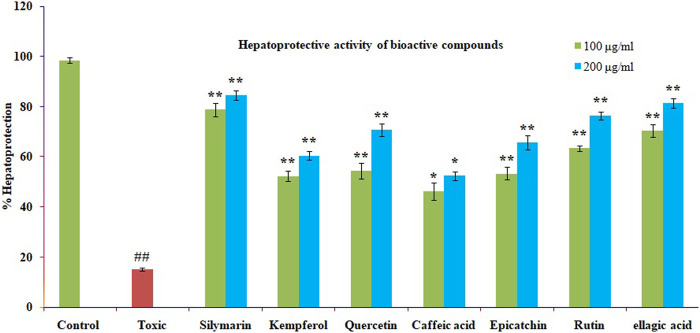
The impact of several isolated bioactive compounds on CCl4-induced toxicity in Chang liver cells. Chang liver cells were treated with CCl4 for 2 h after being cultured for 2 h with various substances in either their presence or absence. The cells were then prepared for the MTT experiment. In comparison to the CCl4 control, the data are expressed as mean S.E.M. with ns *p* > 0.05, **p < 0.01, and *p < 0.05 being significant.

The mentioned bioactive compounds were examined for hepatoprotective effectiveness against CCl4-induced cytotoxicity by pre-incubating the cells with or without the silymarin or extracts. The Chang liver cells’ viability significantly decreased after CCL4 therapy. The results are displayed using the mean standard error mean formula (S.E.M.). The significant high hepatoprotection provided by standard silymarin ranged from 77.6% at 100 μg/ml to 83.95% at 200 μg/ml, purified ellagic acid ranged from 70% at 100 μg/ml to 81.33% at 200 μg/ml, purified rutin ranged from 63.4% at 100 μg/ml to 76.34% at 200 μg/ml purified quercetin ranged from 54.33% at 100 μg/ml to 60.64% at 200 μg/ml, purified catechin ranged from 53.22% at 100 μg/ml to 65.6% at 200 μg/ml, and purified kaempferol ranged from 52.17% at 100 μg/ml to 60.34% at 200 μg/ml, as shown in Figure 7.

## 4 Conclusion

The recommended HPTLC method is unique, as it documents the first time that bioactive compounds in *O. sanctum* (Linn) were identified and quantified using a single solvent system. Feeding experiments were carried out with various concentrations (100 and 200 mg/kg, b w) of alcoholic and aqueous–alcoholic extracts (50%) of selected medicinal plants, and the identification of serum metabolites from the extract-fed rats was carried out using GC-MS. It might be concluded that the metabolites of the serum extract contain a lot of fatty and aliphatic compounds not responsible for hepatoprotective activity. The impact of pretreatment with the alcoholic and hydroalcoholic extracts of *O. sanctum* and silymarin on the biochemical parameters of the rats’ given galactosamine intoxication was examined. According to the findings, the hydroalcoholic extract had a greater hepatoprotective effect than the alcoholic extracts. The hydroalcoholic extract was fractionated into petroleum ether, ethyl acetate, chloroform, and water based on polarity. *O. sanctum*’s ethyl acetate fraction had more hepatoprotective effects than the other fractions. Hence, it was concluded that ethyl acetate is the best fraction for vacuum liquid chromatography. Our research reveals that bioactive compounds extracted from *O. sanctum* provide significant protective benefits against hepatotoxicity caused by galactosamine. This might be a result of the antioxidant and membrane-stabilizing properties of these chemicals. The results show that it is a source of structurally unique bioactive compounds, which may offer possibilities for the creation of novel semi-synthetic molecules for more current indications.

## Data Availability

The original contributions presented in the study are included in the article/Supplementary Material, further inquiries can be directed to the corresponding authors.
